# Nephrolithiasis and Osteomalacia associated with adefovir-induced Fanconi syndrome in a patient with hepatitis B

**DOI:** 10.1186/s12882-017-0693-4

**Published:** 2017-08-29

**Authors:** Jueying Lin, Yufeng Zhuo, Dongdong Zhang

**Affiliations:** Department of Traditional Chinese Medicine, Zhong Shan Hospital Xiamen University, No. 201 Hubin nan Road, Xiamen, Fujian 361004 China

**Keywords:** Adefovir, Renal Fanconi syndrome, Osteomalacia, Osteoporosis, Nephrolithiasis, Obstructive nephropathy

## Abstract

**Background:**

An increasing number of case reports suggest that acquired renal Fanconi syndrome may be associated with prolonged use of adefovir against hepatitis B virus. Renal Fanconi syndrome is an uncommon disease, and its complication with nephrolithiasis is quite rare. Herein, we report a rare coexistence of nephrolithiasis and acquired renal Fanconi syndrome in a chronic hepatitis B-positive patient with prolonged adefovir therapy.

**Case presentation:**

The patient presented with osteomalacia and nephrolithiasis. Consequently, extracorporeal shock-wave lithotripsy and left double-J ureteral stent insertion were considered for obstructive nephropathy, which was caused by nephrolithiasis. However, osteomalacia had been misdiagnosed as osteoporosis before admission to our hospital. On admission, a complexity of multiple fractures, hypophosphataemia, glycosuria without hyperglycaemia and non–anion-gap metabolic acidosis indicated a diagnosis of acquired renal Fanconi syndrome induced by adefovir. After switching from adefovir to entecavir, the patient’s symptoms and laboratory findings improved significantly.

**Conclusions:**

The mechanism responsible for nephrolithiasis in renal Fanconi syndrome is still unclear. We recommend regularly monitoring renal function and serum calcium and serum phosphate to prevent renal Fanconi syndrome during the prolonged use of adefovir for hepatitis B virus.

## Background

Adefovir dipivoxil-induced acute tubular necrosis and Fanconi syndrome have been well known to occur at a dose of 60 mg/d or 120 mg/d for human immunodeficiency virus (HIV) [1, 2]. The first case of acquired Fanconi syndrome associated with prolonged adefovir dipivoxil(10 mg/d) therapy for hepatitis B virus (HBV) had been published in 2008 [3]. Counted by ourselves, more than 40 cases of Fanconi syndrome caused by adefovir dipivoxil therapy for HBV have been reported in PubMed at present.

Fanconi syndrome is a generalized dysfunction of the proximal renal tubules leading to increased loss of amino acids, glucose, sodium, potassium, bicarbonate, calcium and phosphorus in the urine. Osteomalacia is a prevalent complication of Fanconi syndrome which contributes to bone demineralization through acidosis in the blood and urinary wasting of both phosphate and calcium. Hypercalciuria, another characteristic of renal Fanconi syndrome, is arguably the most common risk factor in calcium stone formation, but nephrolithiasis is rather uncommon in Fanconi syndrome [4]. Herein, we describe for the first time a case of nephrolithiasis and osteomalacia associated with adefovir-induced renal Fanconi syndrome.

## Case presentation

A 60-year-old man was referred to our outpatient department for a 30-month history of generalized bone pain without antecedent trauma. He reported no fevers, chills, vomiting, diarrhoea, or other symptoms. The patient had a history of chronic hepatitis B treated with adefovir dipivoxil (10 mg/d) for 60 months and had no family history of nephrolithiasis or metabolic bone disease.

A whole-body bone scan was performed 18 months later in another hospital because of bone pain. It revealed multiple abnormal areas with increased tracer uptake in the bilateral ribs, bilateral sacroiliac joints, and bilateral tibias, which were consistent with multiple fractures. Serum intact parathyroid hormone (PTH) and tumour markers were normal. Osteoporosis was diagnosed and treated with alendronate, calcitriol, and calcium carbonate.

**Fig. 1:**
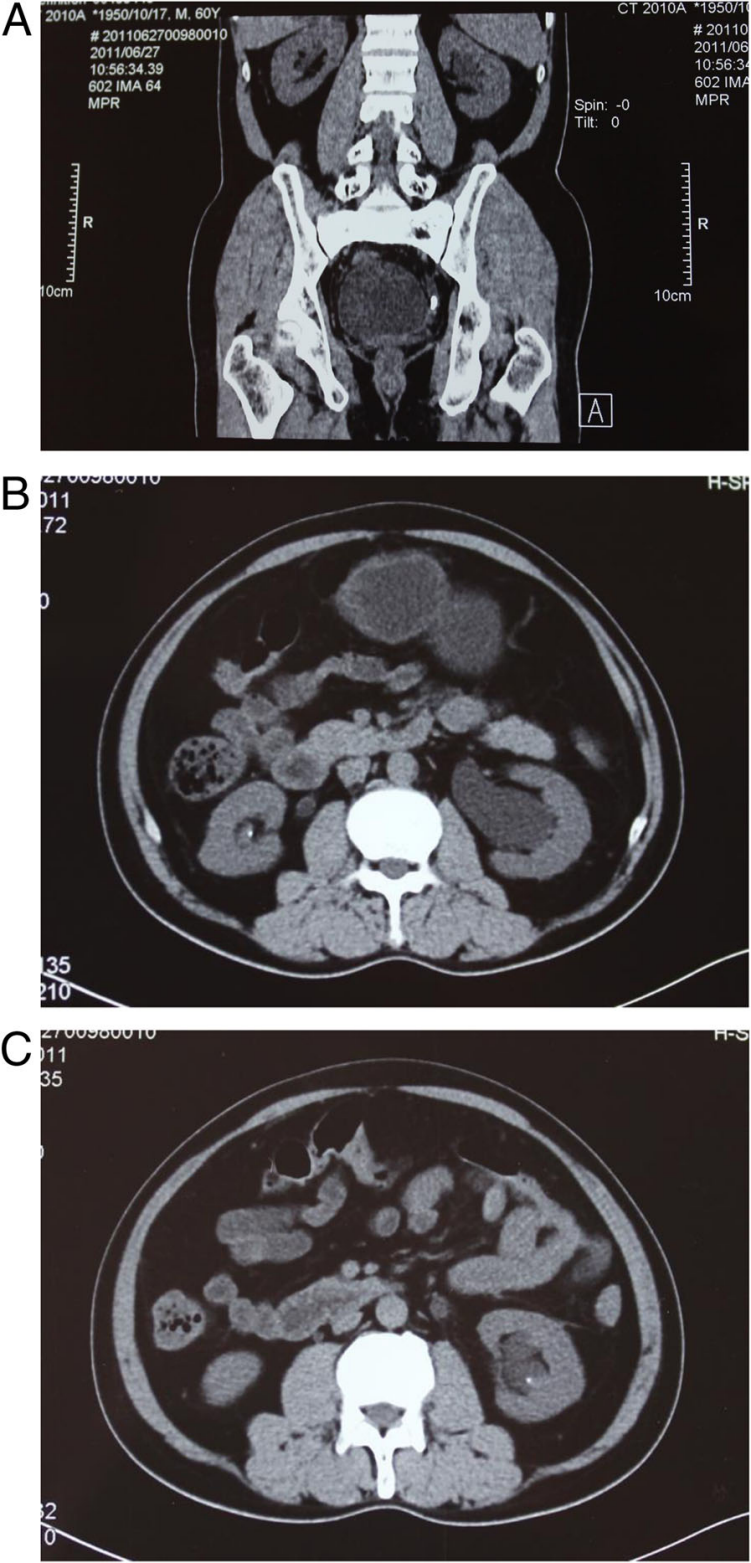
CT imagines. **a**: left ureteral stone combined with left hydronephrosis. **b**: a stone in the right kidney. **c**: a stone in the left kidney

The patient’s bone pain was alleviated slightly after 6 months of treatment. Then, he was sent to the haematology department of the hospital mentioned above. Laboratory data are listed in Table 1. An ultrasound revealed stones in both kidneys and left ureteral stone combined with left hydronephrosis. A further CT scan of the urinary system without contrast revealed high-density images of both renal lower calyces, with left ureteral stone complicated by hydronephrosis on the left kidney, which suggested obstructive nephropathy caused by nephrolithiasis(Fig. 1). The total glomerular filtration rate was 41.89 ml/min evaluated by renal dynamic imaging with 99mTc-DTPA (techne-tium-99 m diethylene triamine penta-acetic acid) as exogenous markers. A DXA scan showed that the T-score of the left femoral neck was −2.60. A re-examination of whole-body 18-FDG-PET/CT showed similar findings as the whole body bone scan performed earlier at another institution. To eliminate bone pain and renal dysfunction caused by multiple myeloma, a bone marrow biopsy was performed, and the results were normal. The cause of renal impairment was presumably nephrolithiasis-induced obstructive nephropathy. Consequently, extracorporeal shock-wave lithotripsy and left double-J ureteral stent insertion were performed to alleviate the nephrolithiasis-induced obstructive nephropathy. Subsequently, alendronate, calcitriol, and calcium carbonate were suspended by the patient for personal reason.

**Table 1:** laboratory Data

Variable	Reference range	Other hospital	On admission	After Treatment
Serum				
Phosphate	0.8–1.6 mmol/l	0.51	0.63	0.94
Calcium	2.17–2.75 mmol/l	2.01	2.12	2.44
Potassium	3.50–5.30 mmol/l	3.38	3.75	3.80
Chloride	96-108 mmol/l	111	107.75	105
Sodium	135-145 mmol/l	142	135	137
Magnesium	0.7–1.10 mmol/l	0.90	0.97	1.01
Urea nitrogen	2.9–8.2 mmol/l	6.04	4.95	5.06
Creatinine	44-133umol/l	158	139.6	134
Uric acid	208-506umol/l	136	133.1	230
Anion-gap	8.0–16.0 mmol/l	9.0	10.7	12
Albumin	35-55 g/l	47.10	45.0	50
ALP	10-150u/l	109	304.9	147
FBG	3.9–6.1 mmol/l	5.7	5.6	5.6
25(OH) D3	27.7–107.0 nmol/l	NA	36.7	NA
iPTH	15-65 pg/ml	22.40	92.29	72.7
Lead(Pb)	0-200μg/l	12.5	NA	NA
Fibroblast growth factor 23	0-180RU/ml	NA	NA	NA
Arterial blood gas				
pH	7.35–7.45	NA	7.32	7.37
AB	21.4–27.3 mmol/l	NA	18.20	22.0
PaCO_2_	4.66–6.00 KPa	NA	4.57	4.86
Urine				
pH	5.0–9.0	6.50	7.00	6.0
Protein	Negative	1+	2+	Negative
Glucose	Negative	2+	4+	Negative
Albumin	4-230 mg/24 h	NA	300	NA
Phosphate	12.9–42.0 mmol/24 h	11.0	56.5	84.9
Calcium	2.5–7.5 mmol/24 h	5.60	0.4	1.8
Uric acid	1.48–4.43 mmol/24 h	NA	3.64	3.77
N-acetyl-glucosaminidase	0.3–12.0u/l	NA	21.0	NA
﻿ β2-MG	0–0.32 mg/l	NA	82.5	NA

ALP-alkaline phosphatase, AB-actual bicarbonate, FBG-fasting blood-glucose, 25(OH)D3–25-hydroxyvitamin D3, β2-MG β2-microglobulin, NA-not available

The patient was then referred to our inpatient department because of bone pain for 30 months. At our first examination, the patient weighed 55 kg, and his height was 168 cm, giving a body mass index (BMI) of 19.5 kg/m^2^. The physical examination showed mild tenderness of the ribs. Laboratory investigations are presented in Table 1. A renal biopsy showed interstitial nephritis, and the immunohistochemistry test for HBV was negative. Mild-to-moderate interstitial nephritis and focal segmental glomerulosclerosis of the renal tissue were diagnosed by electron microscopy. The diagnosis of nephrolithiasis and osteomalacia due to renal Fanconi syndrome caused by adefovir is based on multiple fractures and an elevated level of alkaline phosphatase (ALP), hypophosphataemia due to hyperphosphaturia and non–anion-gap tubular metabolic acidosis accompanied with glycosuria without hyperglycaemia. Consequently, adefovir was substituted by entecavir (0.5 mg/d) against hepatitis B virus, along with the commencement of supplementation with neutral sodium phosphate (1.5 g/d), potassium citrate (4 g/d, divided into three equal parts), calcium carbonate (elemental calcium 0.6 g/d) and calcitriol (0.5 μg/d) orally for osteomalacia. The patient responded dramatically to the entecavir and supplementation therapy, which has now lasted five years.

## Discussion

Osteomalacia is a metabolic bone disorder in adults caused by general defective mineralization of the organic bone matrix. Bone biopsy with double tetracycline labeling is the best procedure to differentiate osteomalacia from osteoporosis [5]. However, it is not easily executed unless the differential diagnosis cannot be made from the medical history. In osteoporosis, normal levels of serum calcium, phosphate, alkaline phosphatase, and PTH are present; by contrast, abnormalities in at least one of these measurements are common in osteomalacia [6]. The bone disorders point to the diagnosis of osteomalacia instead of osteoporosis in the case.

Nephrolithiasis is infrequently encountered in renal Fanconi syndrome [4]. Type 3 renal tubular acidosis is a mixture of distal renal tubular acidosis and renal Fanconi syndrome. It seems more reasonable to explain the coexistence of osteomalacia and nephrolithiasis in this case. During admission to our hospital, secondary hyperparathyroidism appears to counterpoise calcium wasting in the proximal renal tubules by increasing the reabsorption of calcium in the distal tubules. Therefore, the possibility of distal renal tubular acidosis occurring is inadequate.

In theory, the insolubility of adefovir in urinary environment may cause chemical stones. Nephrolithiasis has been demonstrated to be an adverse effect of indinavir when used as an antiretroviral agent for HIV [7]. However, the association between prolonged use of adefovir and nephrolithiasis formation has not been found regardless in basic research or clinical observation [8, 9]. Because extracorporeal shock-wave lithotripsy had been performed for obstructive nephropathy before the patient’s admission to our hospital, we could not precisely identify the character of nephrolithiasis in the patient. Therefore, whether the formation kidney stones directly related to insolubility of adefovir is uncertain in this case.

In the case, calcium stones seem most likely caused by hypercalciuria according to the radio opaque stones in both kidneys. Hypercalciuria was defined in adults as 24-h urine calcium excretion >4 mg/kg of body weight/day [10]. In this case, after adjusting for body weight the value of 24-h urine calcium excretion was 4.07 mg/kg which fulfilled the criteria of hypercalciuria. Hypercalciuria seems to be the major factor contributing to nephrolithiasis in the case. The causes of hypercalciuria in this case were complex. Because calcium reabsorption is defective in Fanconi syndrome, calcium supplementation would aggravate the hypercalciuria [10]. Hypophosphataemia may exacerbate hypercalciuria by increasing the intestinal calcium absorption [10].

Interestingly, mitochondria dysfunction may underline the association between adefovir nephrotoxicity and Fanconi syndrome [11, 12]. Furthermore, a clinical study demonstrated that disruption of the Sodium-inorganic phosphate cotransporter NaPi-IIa (NaPi-IIa) may play a crucial role in the pathogenesis of nephrolithiasis and metabolic bone disease associated with Fanconi syndrome [13, 14]. Therefore, the NaPi-IIa should be a focus of future research regarding adefovir induced Fanconi syndrome.

## Conclusions

It remains a challenge for scientists and clinicians to understand the relationships among adefovir, Fanconi syndrome and nephrolithiasis. Until the mechanisms responsible for adefovir nephrotoxicity are clarified, we recommend regularly monitoring renal function, serum calcium and serum phosphate to prevent renal Fanconi syndrome during the prolonged use of adefovir for HBV particularly for patients with high-risk factors for renal impairment, such as low BMI, old age and decreased GFR.

## Abbreviations

18-FDG-PET/CT: 18F–fluorodeoxyglucose positron emission tomography computed tomography
25(OH)D3: 25-hydroxyvitamin D3
99mTc-DTPA: technetium-99 m diethylene triamine penta-acetic acid
AB: actual bicarbonate
ALP: alkaline phosphatase
BMI: body mass index
DXA: dual energy X-ray absorptiometry
FBG: fasting blood-glucose
HBV: hepatitis B virus
HIV: human immunodeficiency virus
mg/d: milligram/day
NA: not available
NaPi-IIa: Renal sodium-inorganic phosphate cotransporter NaPi-IIa
PTH: parathyroid hormone
β2-MG: β2-microglobulin
